# Effects of Lamellar Keratectomy and Intrastromal Injection of 0.2% Fluconazole on Fungal Keratitis

**DOI:** 10.1155/2015/656027

**Published:** 2015-05-07

**Authors:** Xinying You, Jun Li, Suxia Li, Weiyun Shi

**Affiliations:** ^1^Shandong University, No. 44 Wenhua Xi Road, Jinan 250012, China; ^2^Department of Ophthalmology, Liaocheng People's Hospital, Liaocheng 252000, China; ^3^Shandong Eye Hospital, Shandong Eye Institute, Shandong Academy of Medical Sciences, Jinan 250021, China

## Abstract

*Purpose*. To evaluate effects of lamellar keratectomy and intrastromal injection of 0.2% fluconazole (LKIIF) on fungal keratitis. *Methods*. Data for 54 eyes of consecutive patients with fungal keratitis treated with LKIIF were retrospectively analyzed. The lesions in these eyes did not heal or were aggravated after antifungal chemotherapy for 7 days. The maximum lesion diameters were ≤5 mm and maximum depth was not more than half of full corneal thickness. Cases were followed up for at least 90 days. *Results*. Forty-six eyes were cured (85.2%). The wound healing times were 3–16 days and were less than 7 days in 28 cases (51.9%). In cured eyes, uncorrected visual acuity (UCVA) and best-corrected visual acuity (BCVA) were both 20/250–20/20. The UCVA improved in 38 eyes and was unchanged in seven eyes. BCVA improved in 44 eyes and was unchanged in two eyes. When followed up for more than 90 days, 89% (41 of 46 eyes) showed improvement in UCVA and 11% were unchanged. Regarding BCVA, 98% improved and one eye was unchanged. No other complications were observed except neovascularization in one eye and thinner corneas. *Conclusions*. LKIIF was quick and effective for small fungal keratitis confined to half of the corneal thickness.

## 1. Introduction

Fungal keratitis is one of the most challenging types of infectious keratitis [1], which has been gradually increasing during the past few decades. It now accounts for approximately 50% of infectious corneal diseases [2–4], and corneal blindness has become the second leading cause of blindness in developing countries [5–8]. Fungal keratitis is not easily cured by medication [9, 10]. It usually takes approximately 2-3 months to cure a fungal infection, making it difficult for patients to maintain treatment. Many types of superficial or intermediate fungal keratitis with small diameters are refractory to medication, resulting in eventual corneal transplantation, which is susceptible to surgical complications and usually limited by the lack of donors.

The ultimate goal of fungal keratitis treatment is to preserve or improve visual acuity [11]. To reach this goal, it is important to investigate therapeutic methods that can preserve visual acuity and avoid corneal transplantation. In this study, we evaluated and reported the curative effects of lamellar keratectomy and intrastromal injection of 0.2% fluconazole (LKIIF) on fungal keratitis.

## 2. Patients and Methods

### 2.1. Patients

Fifty-four eyes of 54 consecutive patients admitted to our hospital were analyzed retrospectively. These patients were treated with LKIIF from January 2010 to April 2013 at the Shandong Eye Hospital. The ages of the patients ranged from 21 to 73 years, and the mean age was 46.5 years. Thirty-three patients were men and 21 were women. These patients had no other systemic diseases, except for six patients with diabetes mellitus and four patients with hypertension. No patients had a history of bacterial infections, viral infections, amebic infections, or previous corneal scars. The time from the appearance of symptoms to the first visit was 3−60 days with a mean time of 13.5 days. The best-corrected visual acuity (BCVA) was finger count (FC) to 20/30 (1.83 ± 0.3 LogMAR) at the first visit to the hospital. The patients had used antifungal, antibacterial, and antiviral medications and corticosteroids before their first visit. The diagnosis of fungal keratitis was made if fungal hyphae were found by confocal microscopy, were found in fungal cultures, or were found in corneal scrapings after a wet mount potassium hydroxide procedure. The patient recruitment criteria were as follows: (1) there are invalid or aggravated symptoms, which were considered invalid or aggravated if there was no improvement after antifungal chemotherapeutics for 7 days; (2) location and size of the infected areas of the lesions did not involve the corneal limbus, and the maximum diameters were ≤5 mm; and (3) the maximum depth of the lesion was not more than half of the corresponding corneal full thickness, as determined by slit lamp and anterior segment optical coherence tomography (AS-OCT).

Information about the cornea lesions in our recruited patients is summarized as follows. The maximum diameters of the lesions including ulcers and infiltrations were from 2.0 to 5.0 mm (4.0 ± 0.2 mm). The maximum depths of the lesions were from 24.3% to 52.7% of the corneal full thickness. There were no cases of corneal thinning before surgery, as determined by slit lamp and AS-OCT. The lesions were located in the peripheral cornea (not involving the pupil area) in 20 cases and in the paracentral cornea, but the involved areas covered less than half of the pupillary area in 31 cases, and in the central cornea they covered more than half of the pupillary areas in three cases. Intraocular pressure of all cases was from 13 to 19.5 mmHg. Informed consent was obtained in all cases. Institutional review board/ethics committee approval was obtained from Shandong Eye Hospital Institutional Review Board. The study protocol followed the guidelines of the Declaration of Helsinki.

### 2.2. Antifungal Chemotherapy

All 54 cases received 0.5% fluconazole drops every 15 min and 0.25% amphotericin B or 5% natamycin drops every hour. All drops were used every day until 22:00. Ointment of 1% fluconazole or 1% amphotericin B was applied to the cornea at bedtime. At the same time, all patients received oral itraconazole (0.2 g daily) or an intravenous injection of fluconazole (the first dose was 400 mg followed by 200 mg daily). Two hours after operation, local chemotherapy started again and then gradually reduced, depending on the wound condition.

### 2.3. Surgery and Intrastromal Injection

**Figure 1:**
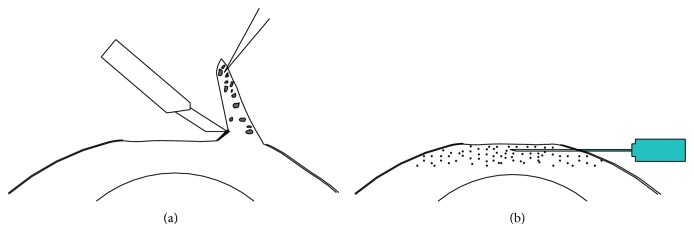
(a) When removing the ulcer, the knife should be kept in parallel with the cornea surface to cut the white fiber between the lesion and the residual cornea. (b) The margin is a slope and the surface is smooth after keratectomy. The injection is from the clear cornea of the lesion edge. The needle is inserted toward the lesion center in parallel with the stroma. The bevel of the needle is in the corneal stroma completely.

**Figure 2:**
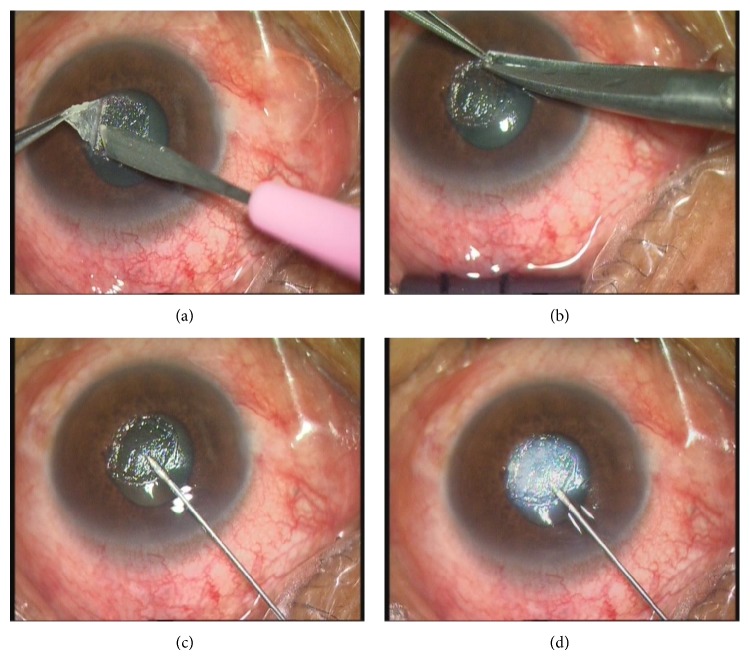
Surgery and intrastromal injection. (a) The corneal flap is held tense with the aid of toothed forceps, and the filamentous white collagen fiber could be seen clearly. (b) Corneal scissors were used to trim the edge to make the edge of wound smooth. (c) The needle was advanced towards the lesion center in parallel with the stroma. (d) Liquid was slowly pushed into the stroma until the inflated stroma exceeded the edge of the lesion by more than 0.5 mm.

All operations were performed under local anesthesia. First, the conjunctival sac and corneal surface were washed with 0.2% fluconazole and the necrotic tissues on the ulcer surface were removed with a corneal epithelium knife. An oblique incision was made 0.5–1.0 mm away from the edge of the lesion. The diseased corneal tissues were gradually moved to the stroma with a blade tilted at 45°. The infected lamellae were pulled up with 0.12 mm forceps, which maintained tension. The blade was held parallel to the stroma to remove the white fiber connecting the lamellae and the stroma. A gentle transition was made at the edge of lesion to the clear area. Next, we washed the wound and inspected the infiltration with an operating microscope. The same procedure was repeated until no residual gray infiltration could be seen. At this point, the lesion including the ulcer and infiltration tissues were completely removed. Corneal scissors were used to trim the edge to make a smooth wound edge. An insulin syringe (1 mL, 30-gauge needle, Becton Dickinson Company, Franklin Lakes, New Jersey, USA) was used for intrastromal injections. The injection was administered from the clear cornea of the lesion edge. The needle was advanced towards the lesion center in parallel with the stroma. When the bevel of the needle was completely in the cornea, 0.2% fluconazole solution was slowly pushed into the stroma until the inflated stroma exceeded the edge of the lesion by more than 0.5 mm. The needle was quickly removed when the injection was completed. This procedure is shown in Figures 1 and 2.

### 2.4. Observations and Statistical Analysis

The corneal infection, healing time of the ulcer, and visual acuity outcomes were observed and recorded. The scars were measured using AS-OCT. Corneal endothelial cells were observed with a corneal endothelial microscope. Intraocular pressure was measured with a Tono-Pen tonometer. Fungal cultures were made from the corneal scraping. If the fungal culture was negative before surgery, another culture was prepared with corneal tissue. Fungal species identification was performed if the fungi culture was positive [12]. The wound healing was defined as a complete coverage of the wound by epithelium. The wound area was checked twice (within 2 weeks) by confocal microscopy and it was considered healed if no fungi were found. The patients were followed for 90 days to 2 years. Statistical analysis was performed using the method of two-tailed independent sample *t*-tests with SPSS 13.0 statistical software (SPSS Inc., Chicago, IL, USA). A *P* value <0.05 was considered statistically significant.

## 3. Results

### 3.1. Clinical Features

**Figure 3:**
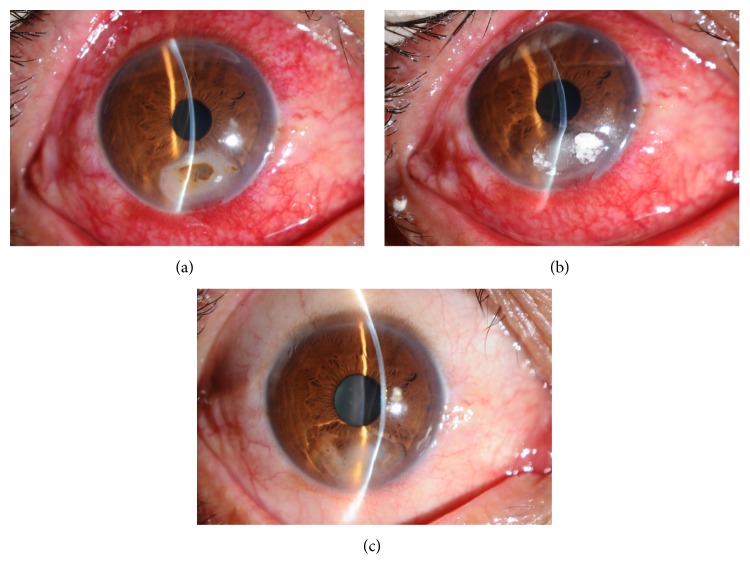
The lesion was located in the peripheral cornea. (a) Before surgery. (b) The first day after surgery. (c) The wound healed 10 days after surgery.

**Figure 4:**
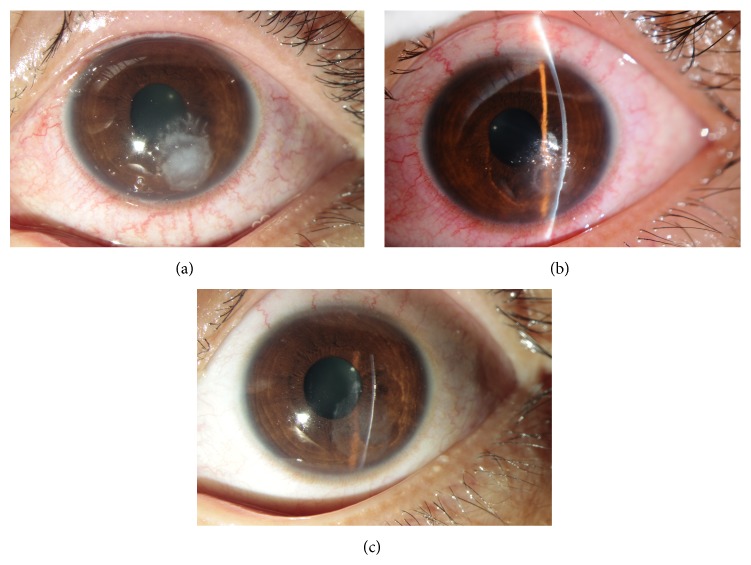
The lesion was located in the paracentral cornea. (a) Before surgery. (b) The first day after surgery. (c) The wound healed 7 days after surgery.

**Figure 5:**
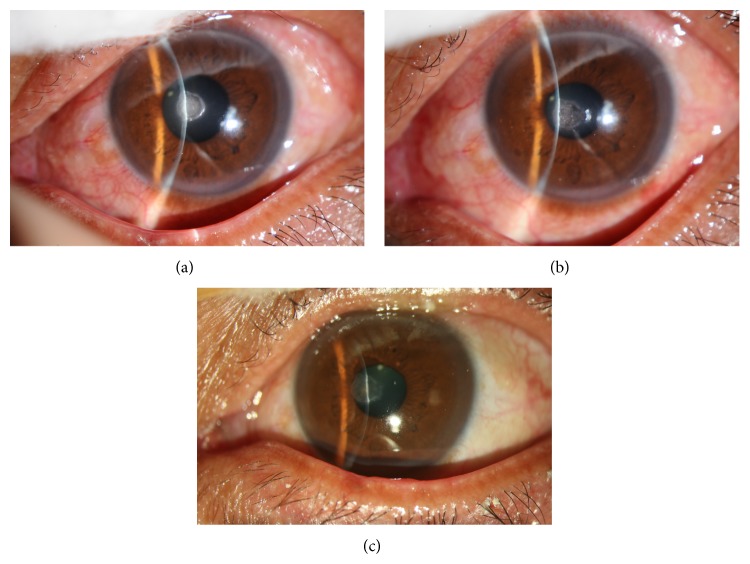
The lesion was located in the central cornea. (a) Before surgery. (b) The first day after surgery. (c) The wound healed 12 days after surgery.

**Figure 6:**
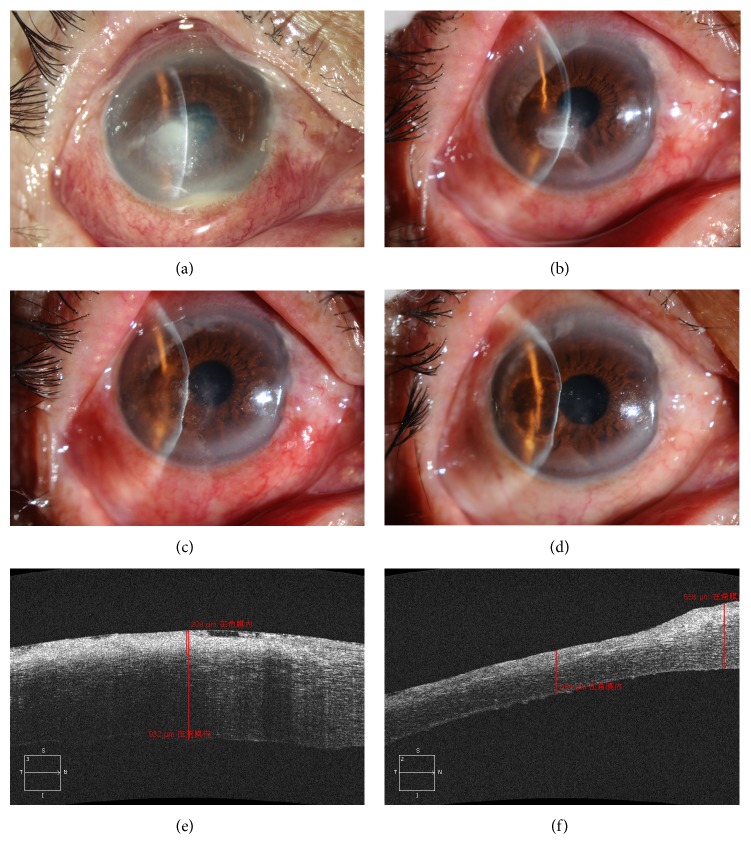
(a) Fungal keratitis with hypopyon. (b) Seven days after antifungal chemotherapy, the hypopyon disappeared but the ulcer had not healed yet. (c) The first day after surgery. (d) The wound healed 7 days after surgery. (e) An OCT image of the lesion before surgery. (f) An OCT image of the lesion when followed up after more than 90 days.

Application of the LFIIK treatment resulted in 46 of the 54 eyes being cured (85.2%) (Figures 3−6). The healing time of the wound was 3−16 days (5.1 ± 1.3), and in 28 cases, the time was less than 7 days (51.9%) with four cases being 3 days. Five cases with hypopyon were cured. However, eight eyes were deemed invalid or aggravated, and these lesions were all located in the paracentral cornea. These wounds did not heal or get smaller. Infiltrations in the stroma were seen in eight eyes, and hypopyon was present in one case. These wounds were covered with white secretions. In contrast, the wounds were very clear and the boundary could be seen clearly in successful cases. Two of the eight eyes had a history of local corticosteroid treatment. For the eight eyes, lamellar keratectomy and inlay conjunctival flap (five eyes), lamellar keratoplasty (two eyes), and penetrating keratoplasty (one eye) were performed, with all eight eyes being cured.

**Figure 7:**
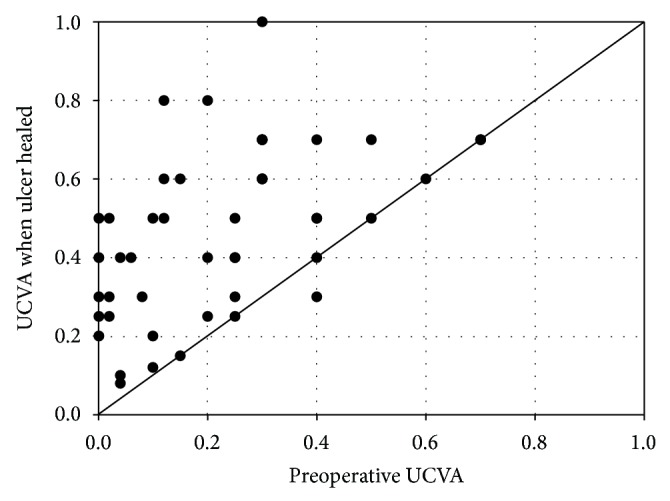
Correlation between preoperative and postoperative UCVA when the wound healed, most of the cases improved their UCVA than preoperation.

We next checked the visual acuities of the cured eyes, and the results are shown in Table 1. The visual acuity of eight eyes in which the treatment failed is shown in Table 2. The uncorrected visual acuity (UCVA) was 20/250–20/20 (0.39 ± 0.24 LogMAR) and was improved in 38 eyes, unchanged in seven eyes, and decreased in one eye (Figure 7). The BCVA was 20/250–20/20 (0.29 ± 0.22 LogMAR) and was improved in 44 eyes and unchanged in two eyes. We followed the patients for at least 90 days and found that the UCVA was 20/250–20/20 (0.36 ± 0.23 LogMAR). Improvement was seen in 41 of 46 eyes (89%) and was unchanged in five eyes (11%). The BCVA was 20/250–20/20 (0.26 ± 0.21 LogMAR), which was an improvement in 45 of 46 eyes (98%) and was unchanged in one eye. The UCVA and BCVA were not statistically different compared with ulcers that were cured (UCVA, *t* = 0.81, *P* > 0.05; BCVA, *t* = 0.55, *P* > 0.05, resp.). There was no statistically significant difference between UCVA and BCVA before surgery. The refractions before the illness were unknown, and postoperative astigmatism was analyzed. Astigmatism after the treatment was recorded as negative values for statistical analysis. If the value was positive, it was changed by converting the axis. The astigmatism was −0.25 to −7.25 D (−2.67 ± 1.84) when ulcers were cured and −0.5 to −7.00 D (−2.67 ± 1.71) when followed up for more than 90 days, which was not statistically different (*t* = 0.015; *P* > 0.05). There were, however, significant differences between BCVA and UCVA when the ulcers were cured (*t* = 2.219; *P* < 0.05). When the ulcers were cured, the UCVA of the cases in the center cornea was 20/80–20/50 and the BCVA was 20/60–20/40. There were no more changes at the follow-up exams.

When followed up for more than 90 days, the depth of the residual cornea scar was 360.5–427.4 *μ*m (373.80 ± 98.73 *μ*m), the maximum diameter of the corneal scar was 3.0–5.0 mm (3.6 ± 0.3 mm), and the depth of the scar was 21.5–49.0 *μ*m (34.30 ± 8.1 *μ*m). The density of endothelial cells was 2865–2377 cells/mm^2^ (2870 ± 321 cells/mm^2^) and the coefficient of variation was 6–18%. The intraocular pressure was 11.0–18.5 mmHg.

### 3.2. Microbiologic Examinations

Corneal scrapings were positive in 46 cases (85.2%). Fungal cultures were positive in 42 cases (77.8%). The results of the species identifications are shown in Table 3. The species of the eight failed cases and the five cases with hypopyon are shown in Tables 2 and 4, respectively.

### 3.3. Complications

Neovascularization was observed in one eye. The cornea in the lesioned area became thinner but no perforation or other complications were found.

## 4. Discussion

Surgeries for fungal keratitis include debridement, inlay conjunctival flaps, lamellar keratectomy, and corneal transplantation [13, 14]. The epithelium in the lesion makes it difficult for antifungal drugs to penetrate the cornea, causing ineffective drug concentrations at the site of the lesion [15]. However, debridement can only remove the epithelium and superficial necrotic tissues. The inlay conjunctival flap has been an effective method for treating refractory keratitis [16, 17], but it works only if the lesion is close to the peripheral cornea [18] and not in the direction of the visual axis that can be covered by the eyelids. Penetrating and lamellar keratoplasty have shown excellent results [19, 20] and are the only procedures available to treat severe cases [20, 21]. However, not all small grafting and eccentric transplantation have resulted in good vision, and graft failure was possible [22]. Furthermore, this approach is also limited owing to a lack of donors; therefore, it is not the first choice for these cases.

The effect of lamellar keratoplasty for fungal keratitis was consistent with the observation that fungi were not always present throughout the whole cornea [20], so keratectomy was feasible [23]. Removing the deeper necrotic tissues and inflammatory agents can directly reduce the damage to tissues caused by inflammation. After keratectomy, the cornea has no vascular system, leading to an insufficient ability to eliminate infections; therefore, adequate use of antifungal drugs is critical for treatment of the remaining fungus. Most antifungal drugs are bacteriostatic agents [24] and have low penetrating ability [25, 26]. However, previous studies showed that intrastromal injection had significant effects [27–30] without inducing tissue toxicity [28, 29]. This procedure is convenient, produces minimal pain, and can achieve higher drug concentrations in lesions compared with eye drops and systemic drugs [31]. However, if intrastromal injection was used alone, the efficiency was limited [32], even with voriconazole, which has a high corneal penetrating ability [26]. Previous studies showed that although newer agents, such as voriconazole, were used in different ways (including intrastromal injection), there are still some cases that required corneal transplantation or enucleation [33, 34]. Therefore, it is better to use lamellar keratectomy. Lamellar keratectomy provided a favorable environment for easy drug penetration into the cornea [31] while the injection maintained high drug concentrations, continuously inhibiting or killing the remaining fungus because of improved bioavailability at the lesion. These findings suggest that the lamellar keratectomy and voriconazole complement each other, which is the most important reason for their success. In the present study, more than half of the cases were cured within a week after surgery with the same local antifungal agents used before surgery. In some cases, the disease was cured within 3 days after surgery. Fluconazole is stable and is a water-soluble small molecule with high bioavailability and low toxicity. It is therefore readily available [25] and suitable for systemic and local use, especially for intrastromal injection.

When the ulcers were cured, the refraction and vision stabilized quickly. The healing time of the wound in our study was 3–16 days, and, in 28 cases, it was less than 7 days (51.9%). In contrast, chemotherapy alone lasted 7 days. UCVA and BCVA in three patients with small graft keratoplasty were lower than the average level of the cured cases in the follow-up. Even if the visual acuity was improved later, it needed more time to improve (Table 2). A previous study showed that chemotherapy for similar refractive fungal keratitis took 28.9 ± 19.1 and 36.1 ± 20.2 days, and the vision after cure was 1.64 ± 0.3 and 1.72 ± 0.23 LogMAR, respectively [32]. Chemotherapy required more than 1 month to cure similar cases caused by* Alternaria* [15], and vision after the cure was 20/15–20/25. Some clinicians have used phototherapeutic keratectomy (PTK) to improve vision for similar cases [35], but this procedure requires special equipment.

**Figure 8:**
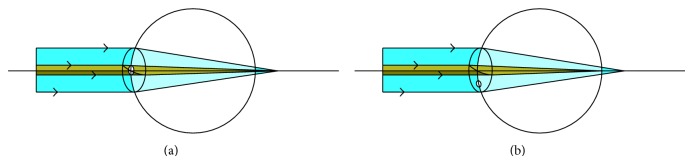
(a) Schematic diagram of the refraction change in an emmetropic eye when a keratectomy is performed in the central cornea. (b) Schematic diagram of the refraction change in an emmetropic eye when a keratectomy is performed in the peripheral cornea.

There are a number of factors affecting vision and refraction after the eye is cured. In theory, keratectomy in the central cornea changes the spherical lens, and peripheral excision changes the cylindrical lens (Figure 8). In reality, both are changed at different levels. The difference of UCVA and BCVA was statistically significant when the ulcer was cured, showing that refractive error is the main factor influencing postoperative UCVA. Astigmatism was observed in some cases after the treatment. Depending on its thickness, area, and location, corneal scarring also had an effect. In this study, corneal scars were thin (34.30 ± 8.1 *μ*m) and their maximum diameters were not more than 5 mm. Keratectomy is not usually performed in the central cornea, to avoid central scarring and a change of refraction. In the present study, three patients whose lesions were located in the central cornea refused keratoplasty for personal reasons and chose keratectomy. The BCVA was 20/200–20/80 when the ulcer was cured while being 20/100–20/40 when followed up for at least 90 days, which was similar to other cured cases. They avoided the complications resulting from keratoplasty and experienced faster vision recovery. However, the indications were more strict, with the maximum lesion depth confined to <40% of the corresponding corneal full thickness, and the maximum diameter was not more than 3 mm.

To cure hypopyon, preoperative antifungal chemotherapy should be used to effectively control the inflammation. Hypopyon is one of the most significant risk factors involving failure [36] and is considered a sign of infection that reaches deep stroma or hyphae, penetrating Descemet's membrane into the anterior chamber. However, the causes of hypopyon could be aseptic or fungal [37]. In our study, the five hypopyon cases were all cured. The healing times and visual acuities were similar to other cases. Case 2 had more hypopyon (4 mm) than case 5 (1 mm) but the healing times for both were identical (Table 4). The results suggest that hypopyon before the surgical procedure is not a sign of severity, an indicator for prognosis, or related to the outcome of keratectomy procedure.* Fusarium* is one of the risk factors for hypopyon [38], which grows horizontally in the cornea [12]. Even if hypopyon occurs, the lesion can only be in the shallow or middle stroma. Most of the patients were infected by* Fusarium,* which may be an important reason for the cure. In addition, intrastromal injection may increase the drug concentration in the anterior chamber.

We believe it is important to carefully inspect the cornea to accurately measure the depth of the lesion before surgery. Use of AS-OCT is increasing in surgical planning and evaluation [39, 40] because it can noninvasively image and measure the profile of each corneal layer. We determined the width and depth of the lesion using the slit lamp first and then used AS-OCT to measure the largest depth. During surgery, it is also important to completely excise the lesion and keep the section smooth to accelerate the growth of epithelium around the wound and to reduce refractive changes. When the corneal flap is holding tension, the white, parallel filamentous fibers could be seen connecting the corneal flap and the stroma. The blade should be held parallel to the stroma to cut the fibers to make a smooth section and gentle transition at the edge of the wound. If the lesion needs to be excised again, this can be initiated from the previous cutting edge on the same plane. To reduce injury from the injection, an insulin syringe was used. The needle was extremely fine and was coated with silicon with the tip polished twice to make it sharper and smoother. The injection should reach the whole lesion and exceed the edge by more than 0.5 mm to make full use of the drug.

Except for neovascularization in one eye, no perforation or other complications were found. Perforation occurred in some cases, excised to not more than half of the corresponding corneal full thickness in PTK, and was considered to be related to the vacuum aspiration during the surgery [35, 41]. Perforation occurred in simple intrastromal injection cases [29, 32], but the injection was not confirmed to be the cause of the perforation. We confined the maximum depth of the lesion to be not more than half of the corresponding corneal full thickness and the maximum diameter of not more than 5 mm. Under these conditions, the difficulty of injection, postoperative instability of the cornea, the amount of remaining fungus, and scarring of the cornea were reduced considerably.

The possible reasons for failure may be attributed to hyphal growth patterns in corneas. In the 54 cases, the pathogenic fungi were* Fusarium*,* Alternaria*, and* Aspergillus* in order of importance, but in previous reports, the order was* Fusarium*,* Aspergillus*, and* Alternaria* [42, 43]. Most* Fusarium* and* Alternaria* grow horizontally in corneas [20]. They cause shallow ulcers, so more cases can be treated with the methodology described in the present report.* Aspergillus* grows vertically in corneas [19], but hyphal growth patterns differ, not only in the same fungal genus but also within the same species [12]. This may be because some of the failed cases were infected by* Fusarium*, with keratoplasty performed for all of the* Aspergillus* cases (Table 2). If infiltration could not be seen under the microscope during surgery then the infiltrated tissue was considered to be removed completely, but failure indicated that there was still visually undetectable fungus present. When the hyphae grew vertically in the cornea such that deeper cornea was involved, it was more difficult to inhibit the remaining fungus by drug treatment. Thus, the location of the lesion was considered as another reason for failed cases. All the failed cases were located in the paracentral cornea. We speculate that the periphery of the cornea adjoined to the corneal limbus and conjunctiva, which have vascular and more anti-inflammatory factors. Few cases were in the central cornea, with different indications making it impossible to be compared with other cases. The type of drug administered may be the third reason for failure. Two cases that received corticosteroids before surgery were failures, and corticosteroids may weaken the ability of cornea to inhibit fungus. In addition, there may be drug-resistant fungus in these patients.

## 5. Conclusions

Our results described a treatment that was effective for the treatment of fungal keratitis not healed or aggravated after general and local fungal chemotherapy. The maximum diameter of lesions, including ulcers and infiltrations, should not be less than 5 mm or not more than 3 mm if the lesion is located in the central cornea. The maximum depth of the lesion should be not more than half of the corresponding corneal full thickness, as determined by slit lamp and AS-OCT, and not more than 40% if the lesion is located in the central cornea. The lesion should not have corneal limbus involvement. In cases of hypopyon, inflammation should be controlled by drugs to quickly remove hypopyon. Surgery was more suitable for fungi growing horizontally in the cornea, and caution should be taken if the fungi grow vertically. The approach described in the present study recovered valuable vision with less damage and fewer complications and needed no donors or special equipment, making it an effective and efficient treatment for fungal keratitis.

## Figures and Tables

**Table 1:** The numbers distribution of BCVA and UCVA (BCVA, UCVA) in 46 cured cases.

Visual acuity	Preoperation	When ulcers were cured	90 days after surgery
<20/400	11, 4	0, 0	0, 0
≥20/400 <20/200	2, 11	1, 0	1, 0
≥20/200 <20/40	27, 22	21, 20	16, 13
≥20/40 <20/25	6, 9	21, 23	24, 27
≥20/25 <20/20	0, 0	3, 3	5, 6

**Table 2:** Clinical characteristics of 8 failed cases.

Patient	The second surgery	The max diameter of lesion^∗^ (mm)	The max depth of lesion^∗^ (%)	Fungus identified	UCVA^†^	BCVA^†^
1	LKT + CF	4.0, 4.0	41.5, 37.3	*F. solani *	20/60, 20/80	20/60, 20/60
2	LKT + CF	3.5, 3.5	30.6, 53.7	*F. solani *	FC/BE, 20/100	FC/BE, 20/80
3	LKT + CF	4.0, 4.5	49.2, 50.0	Negative	FC/1.5 m, 20/60	FC/1.5 m, 20/60
4	LKT + CF	5.0, 5.0	46.5,49.5	Negative	20/125, 20/80	20/125, 20/80
5	LKT + CF	4.5, 5.0	43.5, 40.5	*F. solani *	20/100, 20/80	20/100, 20/60
6	LKP	4.0, 5.0	45.0, 70.0	*A. versicolor *	20/200, 20/160, 20/80	20/200, 20/160, 20/60
7	LKP	5.0, 5.5	40.2, 63.0	*A. flavus *	20/200, 20/100, 20/100	20/100, 20/100, 20/80
8	PKP	4.5, 5.0	39.4, 92.6	*A. fumigatus *	FC/1 m, 20/200, 20/60	FC/1 m, 20/100, 20/40

^∗^The clinical features before the first and the second surgery (first, second).

^†^BCVA values are before the first surgery and 90 days after the second surgery and when followed up for 1 year in the keratoplasty cases (first, second).

LKT + CF, lamellar keratectomy and inlay conjunctival flap; LKP, lamellar keratoplasty; PKP, penetrating keratoplasty; FC, finger count; BE, before eye.

**Table 3:** Fungal species identified in the 38 positive cases in fungal culture.

Fungus	Number of cases	%
Fusarium genu*s *	23	54.8
*F. solani *	7	16.7
*F. moniliforme *	8	19.0
*F. oxysporum *	1	2.4
*F. subglutinans *	3	7.1
*F. sporotrichioides *	2	4.8
*F. semitectum *	1	2.4
Unspecified	1	2.4
*Alternaria* genus	9	21.4
*Bipolaris* genus	1	2.4
*B. hawaiiensis *	1	2.4
*Aspergillus* genus	7	16.7
*A. versicolor *	2	4.8
*A. fumigatus *	3	7.1
*A. flavus *	2	4.8
*Scopulariopsis* genus	1	2.4
*Curvularia lunata* genus	1	2.4
Total	42	100

**Table 4:** Clinical characteristics of all 5 hypopyon cases.

Patient	Hypopyon height (mm)	Max diameter of lesion (mm)	Max depth of lesion (%)	Healing time (days)	Fungus identified	UCVA^∗^	BCVA^∗^
1	1	3.5	38.0	8	*F. solani *	20/200, 20/60	20/200, 20/40
2	4	2.5	37.0	9	Negative	FC/20 cm, 20/60	FC/20 cm, 20/60
3	1	3.0	28.5	7	*F. moniliforme *	20/160, 20/40	20/160, 20/25
4	3	5.0	22.3	3	*F. solani *	FC/BE, 20/80	FC/BE, 20/40
5	1	5.0	45.0	9	*F. solani *	FC/BE, 20/100	FC/BE, 20/60

^∗^UCVA and BCVA are before the first surgery and 90 days after surgery (before, after).
